# Supplementary material for: PIKfyve controls dendritic cell function and tumor immunity
Source: bioRxiv. 2024 Jul 1:2024.02.28.582543. Originally published 2024 Mar 2. Preprint. [Version 2] doi: 10.1101/2024.02.28.582543 (PMC10925294; doi:10.1101/2024.02.28.582543)

Extended Data Figure 1

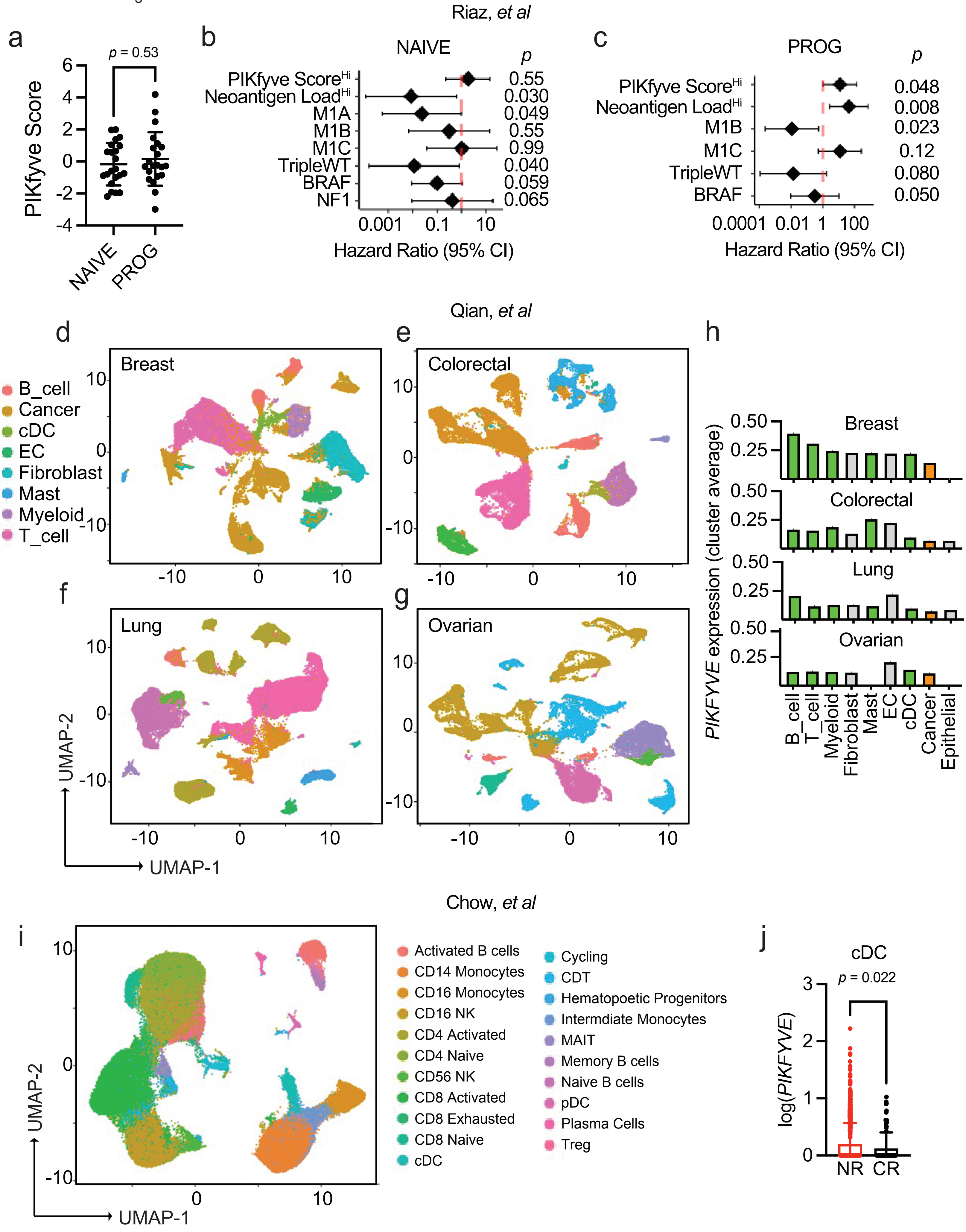

Extended Data Figure 2

a

Gating strategy for Supp 2b-g

**Subset**	**Gating Strategy**
Naïve	$CD3^{+}CD8^{+}CD44^{Lo}CD62L^{+}$
Terminally differentiated	$CD3^{+}CD8^{+}CD44^{Hi}KLRG1+$
Resident Memory	$CD3^{+}CD8^{+}CD44^{Hi}KLRG1^{-}CD49a+$
Exhausted	$CD3^{+}CD8^{+}CD44^{Hi}KLRG1\text{-}CD49a\text{-}TIM3^{+}PD\text{-}1^{+}$
Central memory	$CD3^{+}CD8^{+}CD44^{Hi}KLRG1\text{-}CD49a\text{-}TIM3^{-}PD\text{-}1^{-}CD62L^{+}$
$CD69^{+}$ Effector	$CD3^{+}CD8^{+}CD44^{Hi}KLRG1\text{-}CD49a\text{-}TIM3^{-}PD\text{-}1^{-}CD62L^{-}CD69^{+}$
$CD69^{-}$ Effector	$CD3^{+}CD8^{+}CD44^{Hi}KLRG1\text{-}CD49a\text{-}TIM3^{-}PD\text{-}1^{-}CD62L^{-}CD69^{-}$

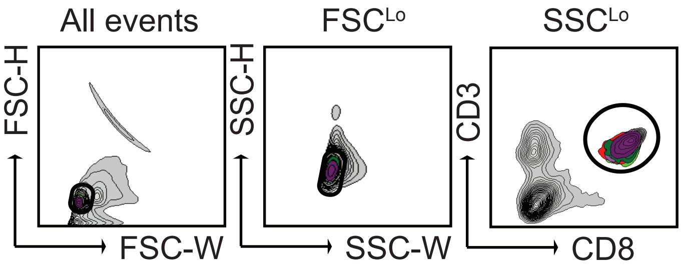

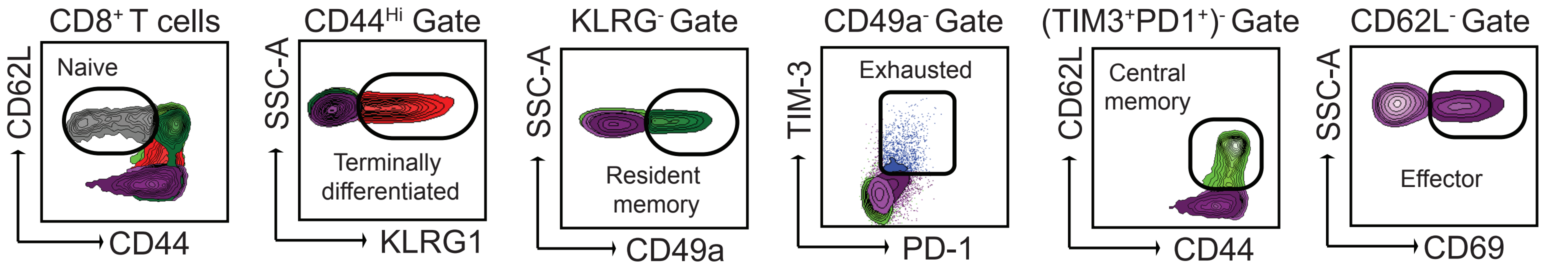

b c d e f g

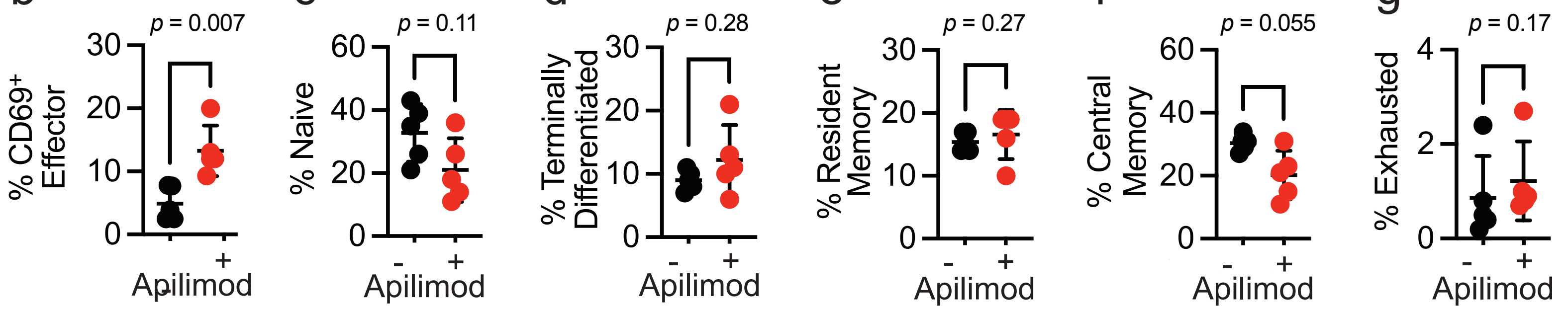

h

Gating strategy for Fig.2a-b, Supp 2i-l

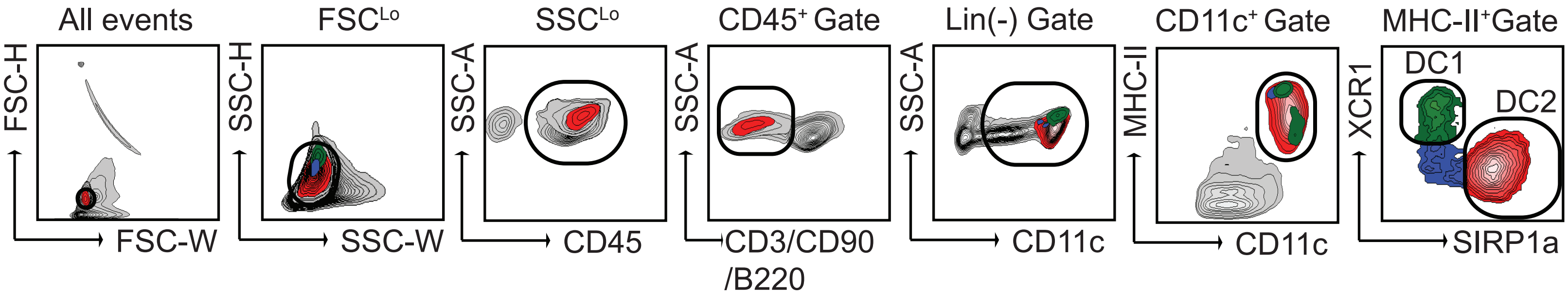

i j k l

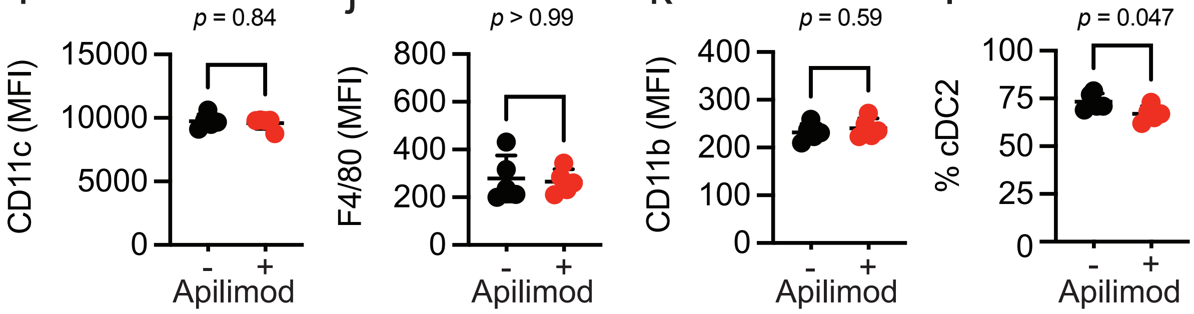

Extended Data Figure 3

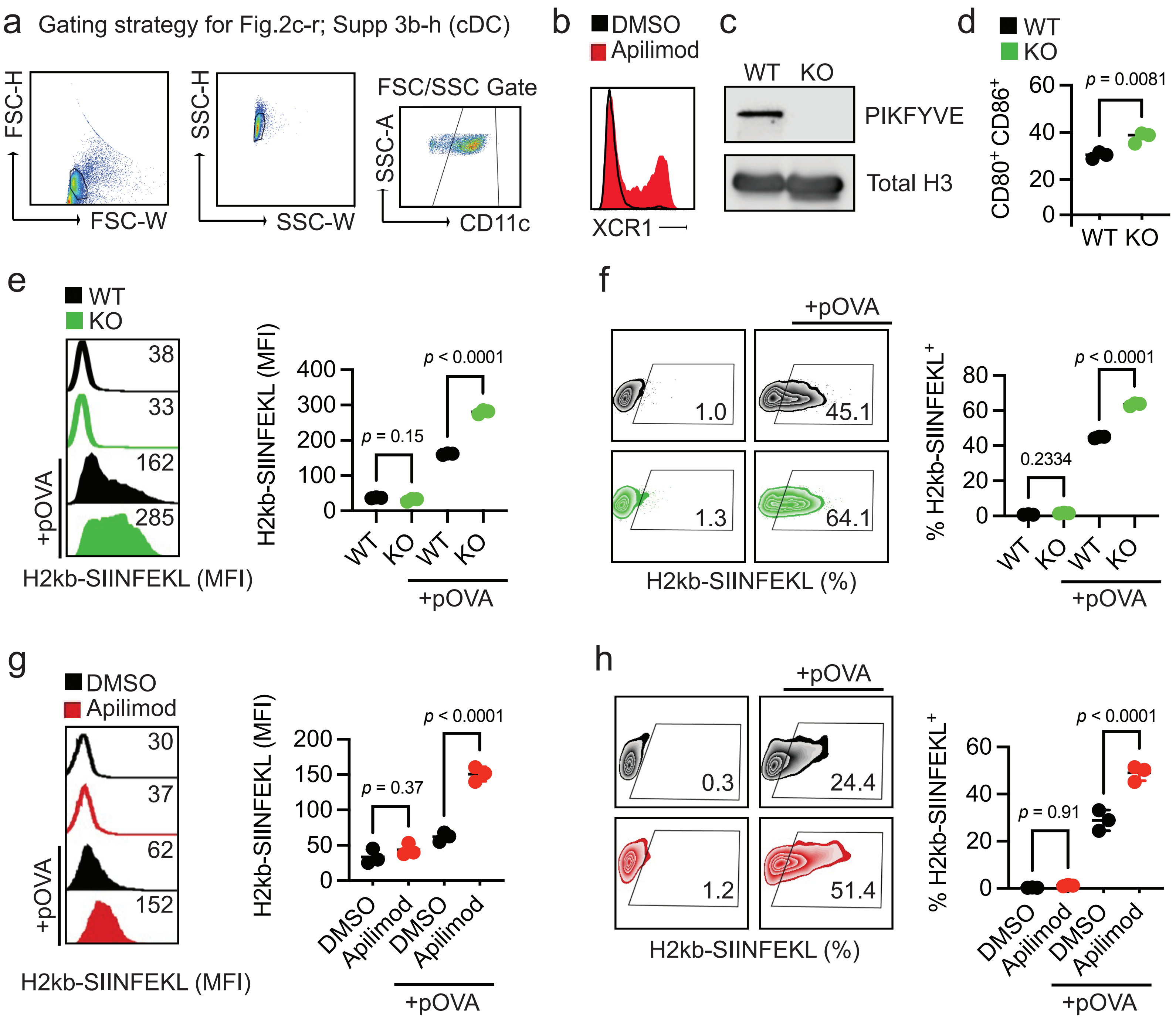

Extended Data Figure 4

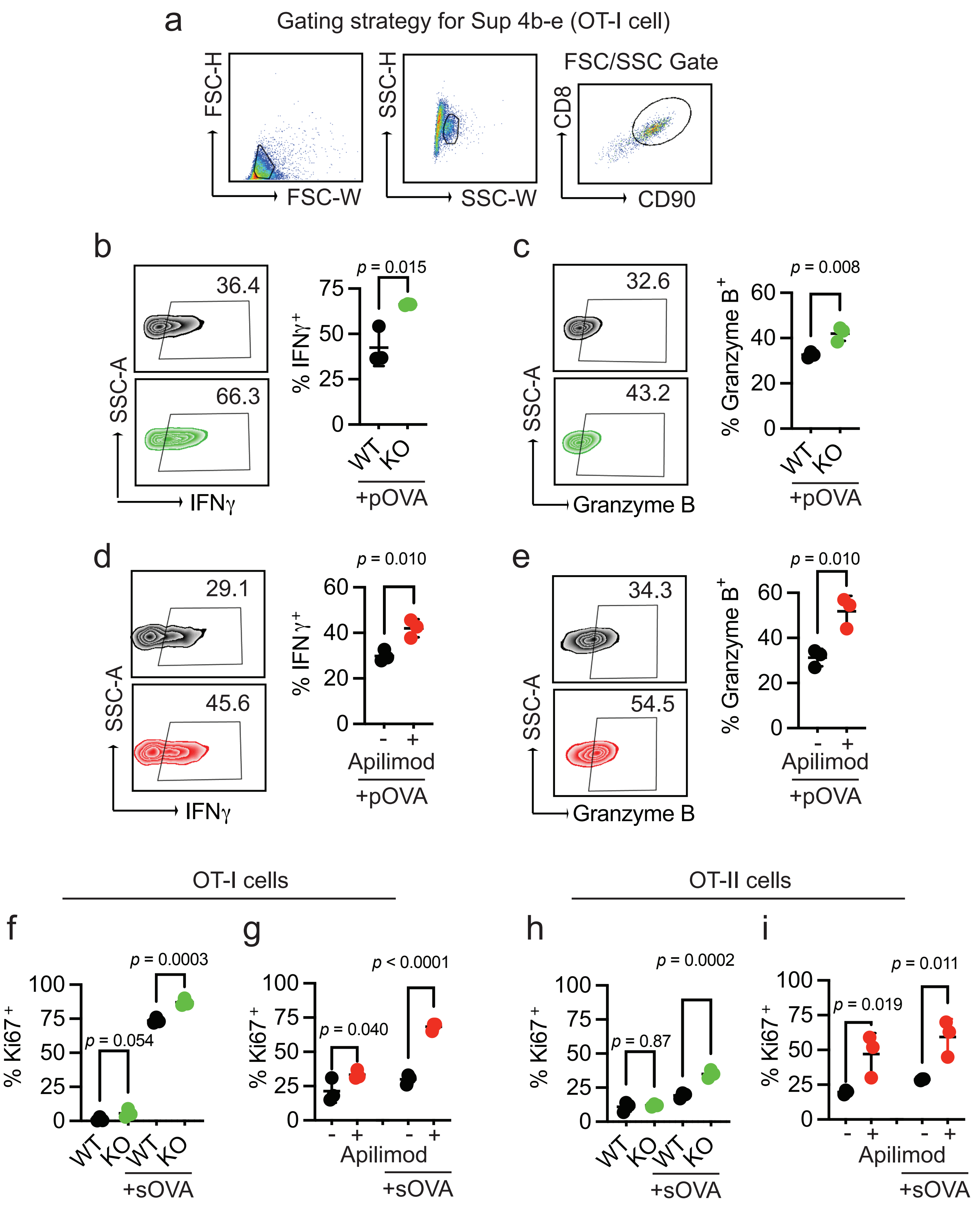

Extended Data Figure 5

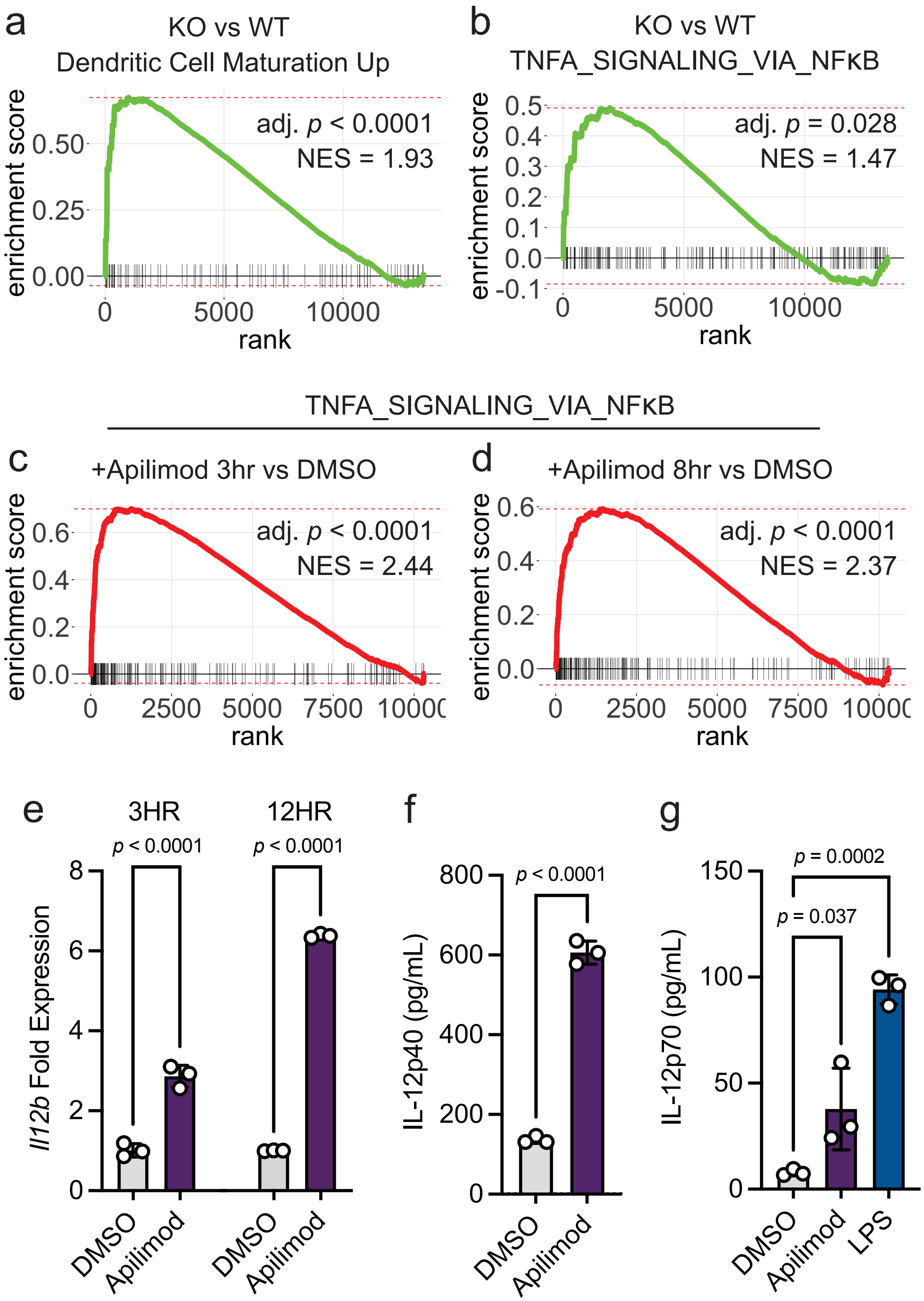

Extended Data Figure 6

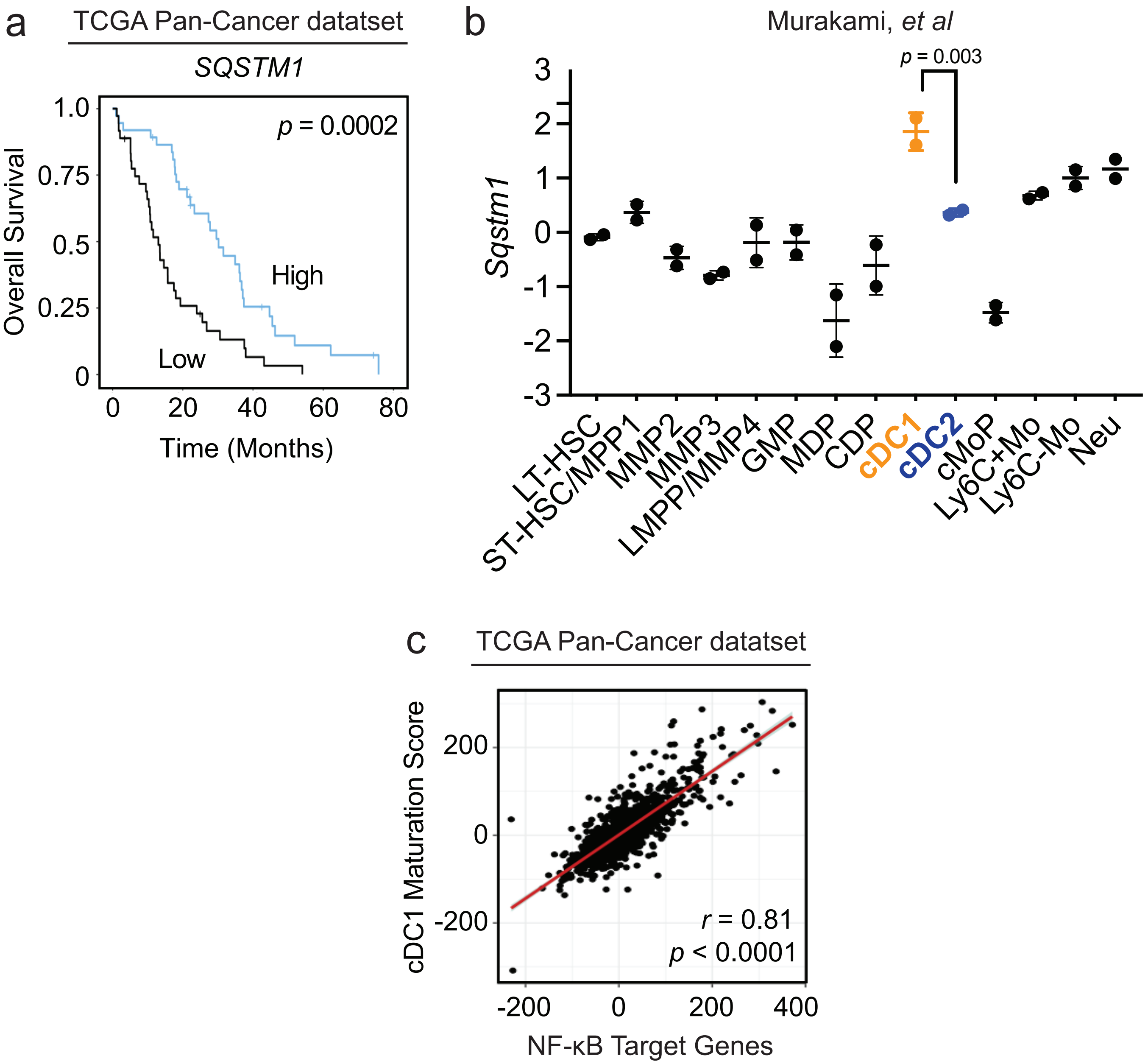

Extended Data Figure 7

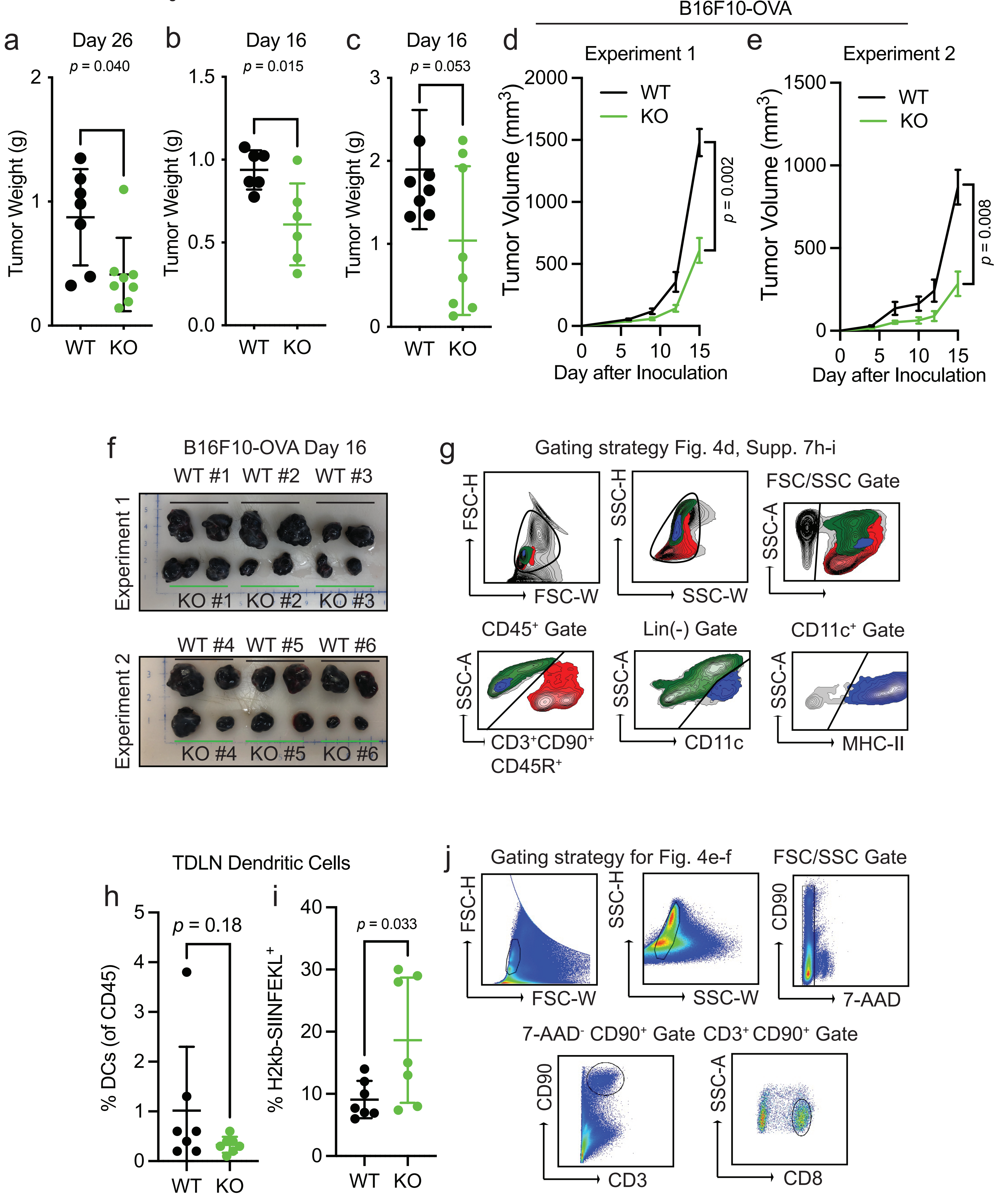

Extended Data Figure 8

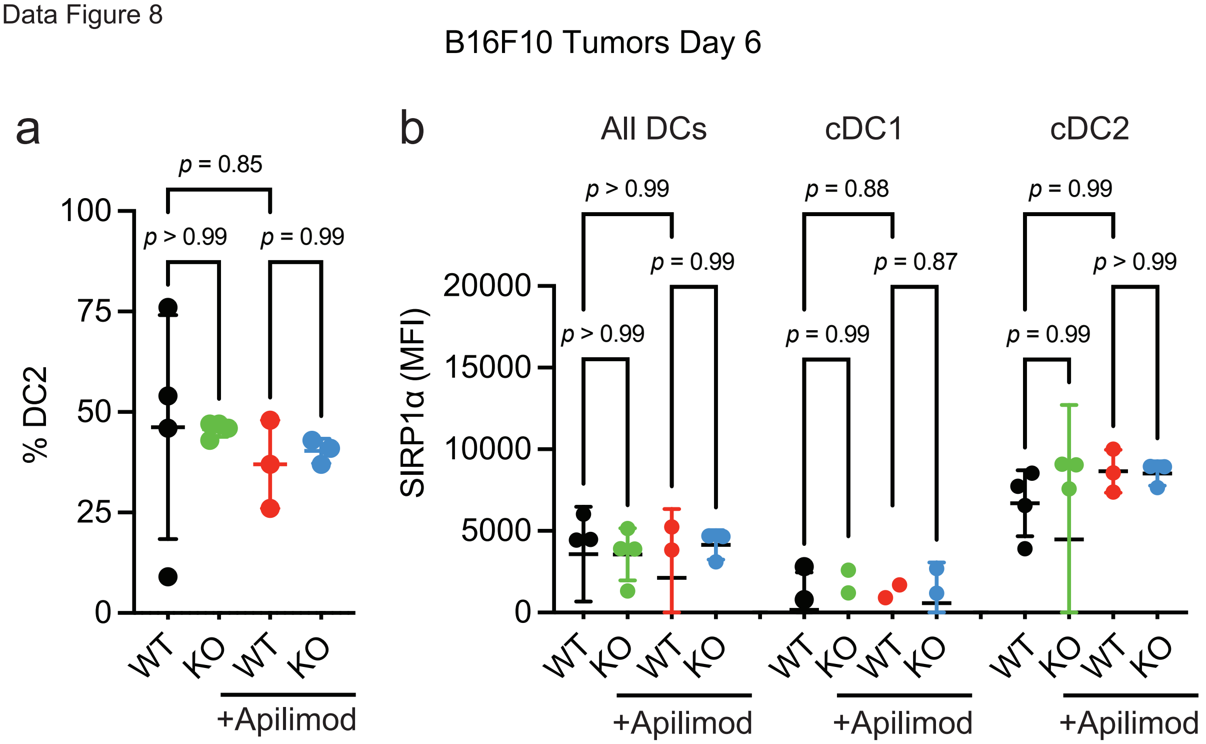

Extended Data Figure 9

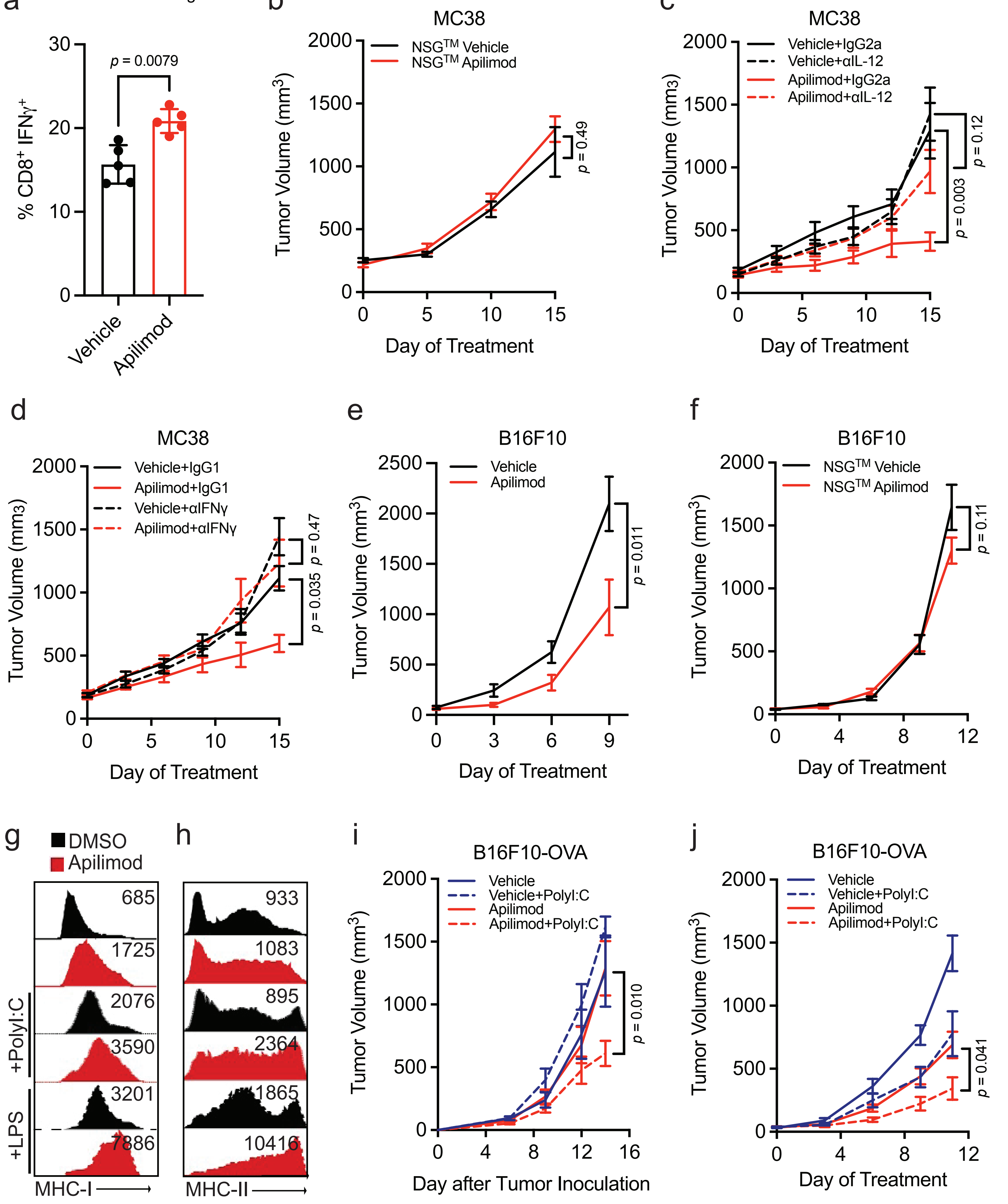

Supplement: Supplement 1 — Extended Data Fig. 1 Supp 1a. Comparison of PIKfyve scores in patients with melanoma who were ICB treatment-naïve (“NAÏVE”) versus those who had previously progressed on ICB treatment (“PROG”). Data plotted are mean ± s.d. from bulk RNA-seq data.44 P value is determined by Mann Whitney U test. Supp 1b. Forest plot of hazard ratios of NAÏVE cohort by high vs. low PIKfyve score, high vs. low neoantigen load, disease stage (reference is M0), and mutation subtype (reference is None). Data plotted are hazard ratios with 95% confidence intervals from bulk RNA-seq data.44 P values are determined by a multivariate cox proportional hazards model. Supp 1c. Forest plot of hazard ratios of PROG cohort by high vs. low PIKfyve score, high vs. low neoantigen load, disease stage (reference is M1A), and mutation subtype (reference is None). Data plotted are hazard ratios with 95% confidence intervals from bulk RNA-seq data.44 P values are determined by a multivariate cox proportional hazards model. Supp 1d-g. t-SNE plots of immune cells in pre-treatment tumors from patients with (d) breast, (e) colorectal, (f) lung or (g) ovarian cancer from scRNA-seq data.48 Supp 1h. Barplots of PIKFYVE cluster average expression by cancer, stroma, or immune cell type from scRNA-seq data.48 Supp 1i. t-SNE plot of immune cells in pre-treatment tumors from patients with endometrial cancer from scRNA-seq data.50 Supp 1j. Comparison of log (PIKFYVE) expression in cDCs in patients with endometrial cancer who were nonresponders (“NR” including SD and PD) versus CR to ICB treatment. Data plotted are mean ± s.d. from scRNA-seq data.50 P value is determined by student t-test with Welch’s correction. Extended Data Fig. 2 Supp 2a. Gating strategy for Supp 2 panels b-g for CD8+ T cells. Supp 2b-g. Comparison of the percentage of (b) CD69+ effector, (c) naïve, (d) terminally differentiated, (e) resident memory, (f) central memory, and (g) exhausted CD8+ T cells from spleens of non-tumor-bearing mice treated wit [file media-1.pdf]
